# Hand-Me-Down Hazard: Flame Retardants in Discarded Foam Products

**DOI:** 10.1289/ehp.123-A56

**Published:** 2015-03-01

**Authors:** Kellyn S. Betts

**Affiliations:** Kellyn S. Betts writes about environmental contaminants, hazards, and technology for solving environmental problems for publications including *EHP* and *Environmental Science & Technology.*

On 1 January 2015 California implemented the first U.S. rule mandating that certain products containing polyurethane foam be labeled to identify whether they contain chemical flame retardants.^1^ Furniture industry experts predict flame-retardant-free couches, chairs, and other padded furnishings and products will be popular with consumers and large purchasers,^2^ and the new labeling law, known as SB 1019,^1^ is expected to have influence beyond the state’s borders, just as California’s flammability standard once drove the use of flame retardants in the rest of the country, and even other parts of the world.^3^ Crate and Barrel, IKEA, and La-Z-Boy are among the manufacturers that reportedly offer or will offer furniture with no added flame retardants.^4^

**Figure:**
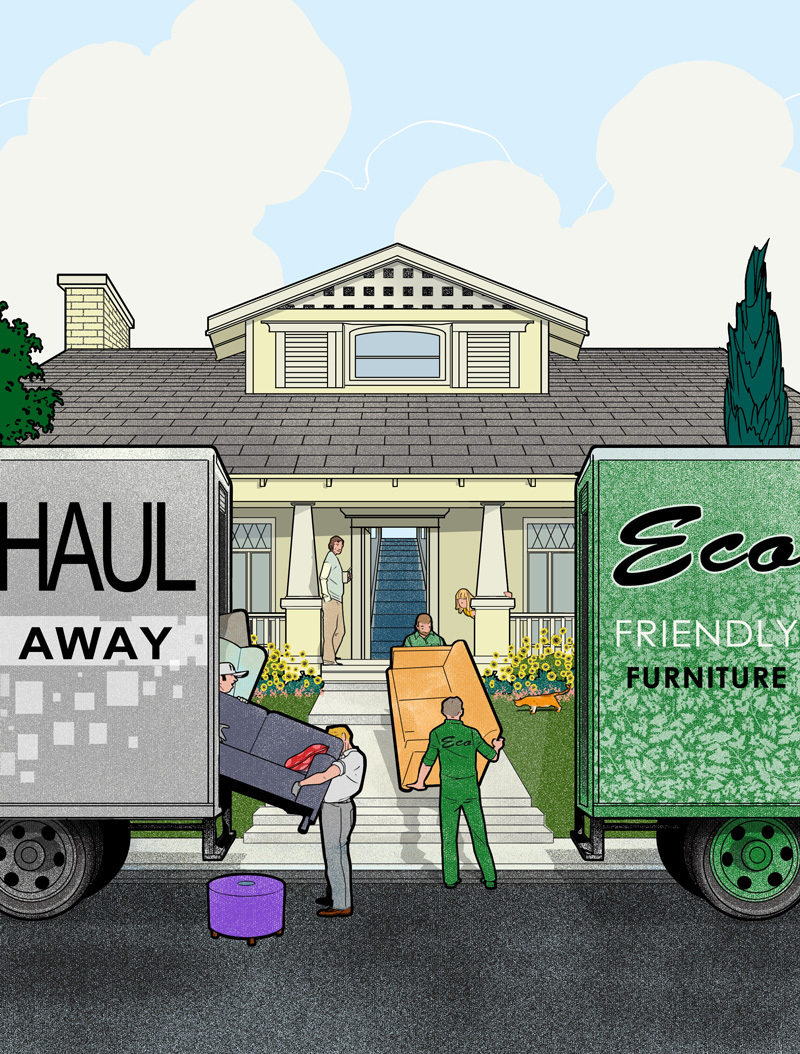
New furniture labeling and flammability standards in California are expected to offer a market-driven solution for people who want to avoid flame retardants. © Jason Schneider

Environmental chemists, scientists, and public health specialists interviewed for this article agree that the new labeling rule represents a great leap forward for consumers. “The consumer should always have the right to know what’s in their products, whether they’re commercial products, food, or anything else,” says Linda Birnbaum, director of the National Institute of Environmental Health Sciences.

Essentially, California’s new labeling rule, and the updated flammability standard that supports it, known as TB117-2013, create one market-based solution to the U.S. problem of widespread exposure to flame retardants while maintaining fire safety. With these new rules, consumers who wish to avoid flame retardants have an option for doing so. Over time, the rules are expected to slowly reduce the health risk posed by human exposure to flame retardants used in polyurethane foam.^5^

However, the benefits may not apply equally to all populations; the ways that discarded furniture and other foam products are handled may disproportionately influence the flame retardant exposures and health of people in disadvantaged communities. Furthermore, how these items are handled can affect the amounts of flame retardants that escape into the environment.

As of now, there are no rules or requirements that address these issues federally or in California, according to officials from California’s State Water Resources Control Board, Department of Toxic Substances Control, and CalRecycle, the state’s recycling agency. Monitoring or control of flame retardants in the outdoor environment is not on the radar of many people outside the research community, says Mark La Guardia, a senior environmental scientist at the Virginia Institute of Marine Science. But he and others interviewed for this article argue that it should be.

## The Need for Fire Safety

Polyurethane foam has many qualities that make it attractive for products that need cushioning. It is lightweight, quiet, low in odor, and resistant to mildew and other triggers of common allergies, and it can be easily molded and cut.^6^

But it is also flammable.^7^ For nearly 40 years, furniture manufacturers added flame retardants to polyurethane foam so it would pass the open-flame test required by California’s original flammability standard, TB117.^8^ In the decades between 1970 and 2004 approximately 46,000 metric tons of PentaBDE, a commercial mixture of polybrominated diphenyl ethers (PBDEs), were used in the United States and Canada, mainly in furniture.^9^ Flame retardants were used in sufficiently high quantities that even after more than two decades, the PentaBDE in one old couch accounted for approximately 4% by weight of foam samples taken from its cushions, according to Heather Stapleton, the Duke University environmental analytical chemist who tested them.

Due to growing concerns about adverse risks associated with PentaBDE constituents (congeners), production of the commercial product was voluntarily phased out by U.S. industry in 2004^10^ and banned by the European Union the same year.^11^ In 2009 it was banned by the Stockholm Convention on Persistent Organic Pollutants, which the United States has signed but not ratified.^12^

Other findings indicate that added flame retardants may not provide the assumed level of safety after all. Recent testing by the Consumer Product Safety Commission found that flame retardants performed inconsistently in deterring the spread of fire.^13^^,^^14^ An award-winning series by reporter Michael Hawthorne for the *Chicago Tribune* revealed that some of the claims used to support the use of chemical flame retardants could not be verified.^15^

Whereas TB117 required that cushions withstand both open-flame and smolder tests, TB117-2013 focuses on the ability of upholstery fabric to pass an updated smolder test. Manufacturers can meet the new standard without the use of chemical flame retardants, but if flame retardants are used, they must be declared on the product label.^1^

**Figure:**
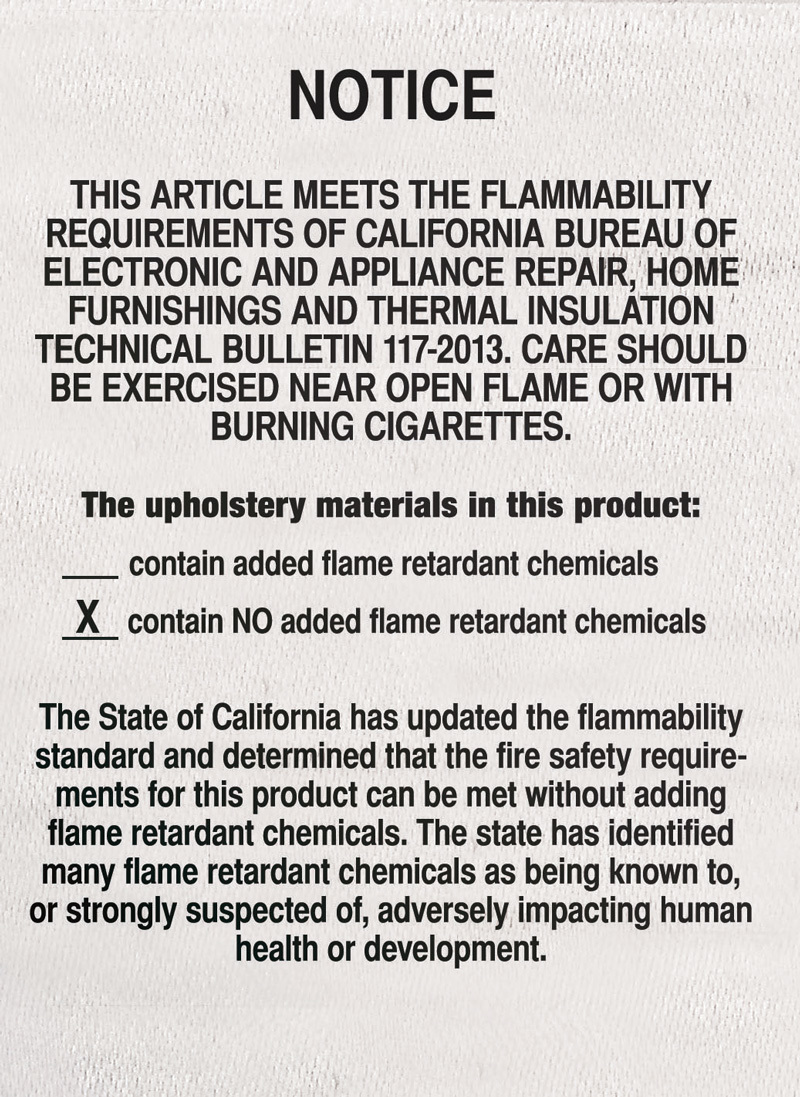
Under California’s new rule SB 1019, any foam product that is subject to flammability testing under TB117-2013 must also carry a permanently attached label that states whether flame retardant chemicals have been added to the item. The rule applies to products manufactured and sold in California after 1 January 2015. © Joseph Tart/EHP

The new standard also increases the number of infant products exempted from testing for compliance—in other words, products that don’t need to demonstrate flame resistance. Under the new standard, bassinets, booster seats, car seats, changing pads, floor play mats, high chairs, high chair pads, bouncers, carriers, mattresses, mattress pads, infant seats, infant swings, walkers, nursing pads, nursing pillows, playpen side pads, play yards, portable hook-on chairs, and strollers now are all exempt from testing.^16^ This is significant because a recent study of 40 childcare facilities in California showed that some children were exposed to PentaBDE and other flame retardants at levels that exceed what the state considers safe daily intake.^17^

Time will tell how the new standard affects rates of fire occurrence and survival. However, the popularity of smoking—historically a major cause of residential fires—continues to decline, and burning cigarettes are now designed to go out if left unattended.^18^^,^^19^^,^^20^

## Flame Retardant Exposures

Hundreds of millions of padded items containing flame retardants are currently in use in the United States, estimates Arlene Blum of the Green Science Policy Institute. That estimate doesn’t include the many hundreds of millions more items already in landfills. Blum and her organization spent eight years campaigning for the changes in California’s flammability standards while also urging lawmakers to find a good way to dispose of older items that contain treated foam.

Because flame retardants are not chemically bonded to the foam, they are able to escape into the surrounding environment.^21^ Indoor air and dust are thus major sources of exposure to some flame retardants.^22^ The National Toxicology Program is currently assessing specific PentaBDE congeners for carcinogenicity,^23^ and peer review is planned for June 2015. Multiple prospective U.S. birth cohort studies have reported a 4.5- to 5.5-point decrement in IQ for each 10-fold increase in PBDE body burden.^24^^,^^25^^,^^26^ Other flame retardants found in newer furniture include tris (1,3-dichloropropyl) phosphate (TDCPP), which is on California’s Proposition 65 list of substances known to cause cancer or reproductive harm.^27^ Another flame retardant mixture known as Firemaster 550_®_ has been associated with obesity, anxiety, and developmental problems in *in vitro* and animal studies.^28^^,^^29^ Little toxicity information is available for still another chemical used as a flame retardant in foam, 2,2-bis(chloromethyl)propane-1,3-diyltetrakis(2-chloroethyl) bisphosphate.

Components of these flame retardants have been found in homes, cars, and foam samples taken from baby products.^30^^,^^31^ Several studies suggest that children receive greater exposures than adults, with one study detecting the major metabolite of TDCPP in children at levels nearly five times higher than in their mothers.^26^^,^^32^^,^^33^ Data collected via the National Health and Nutrition Examination Survey (NHANES) indicates that PentaBDE congeners are present in the blood of virtually all Americans and at higher levels in children than adults.^34^^,^^35^

North Americans’ levels of PentaBDE congeners are significantly higher than levels in Europeans or Asians largely because the product was essentially made and used only in North America as the domestic market sought to comply with TB117.^36^ Within North America, sampling suggests that Californians have the highest average concentrations of PBDEs in their homes and their bodies, once again possibly a result of the state’s flammability standards.^37^

Research conducted over the past decade has shown that socioeconomically disadvantaged communities and people of color may be disproportionately exposed to flame retardants indoors. These studies, which have relied on nationally representative NHANES data, showed that non-white Californians living below the poverty level had some of the highest levels of PentaBDE congeners ever documented.^37^^,^^38^^,^^39^^,^^40^ More recently, studies carried out on opposite sides of the country reported that lower maternal educational attainment (California) and lower socioeconomic status (North Carolina) were associated with higher levels of some PentaBDE congeners and other flame retardants in children.^41^^,^^42^ Other work links lower body burdens of PBDEs in an ethnically diverse population of 6- to 8-year-old girls in California and Ohio with higher-educated caregivers.^43^ This study also found the highest body burdens among black girls.Researchers have also found that low-income residences tend to have higher levels of flame retardants in dust.^44^ Little has been published on levels of newer flame retardants in the bodies or homes of lower-income populations.

Few hard data exist to explain these disparities in flame retardant exposures, says Ami Zota, an assistant professor at George Washington University’s Department of Environmental and Occupational Health. It is possible that the physical weathering and crumbling of treated foam in older or cheaper furniture, which is more often found in lower-income homes, may release greater amounts of flame retardants into indoor environments.^45^ Housing quality, ventilation rates, and the number of residents per square foot may also play a role, Zota says.

Research to date suggests the withdrawal of PentaBDE from the market a decade ago is having a positive impact on human body burdens of the chemical. In 2011 a study of 36 pregnant California women showed their average lipid concentration of PentaBDE congeners was 39% lower than the average lipid concentrations measured in a similar group of women three years earlier.^5^ Both groups were recruited from the same clinic, which primarily served low-income communities, and the earlier group’s levels had been among the highest ever reported for pregnant women, says Zota, a study coauthor.

But some investigators are concerned that the benefits of a market-based approach to removing flame retardants from furniture and baby products will trickle down most slowly to the economically disadvantaged, setting them further behind as far as their chemical body burden goes. “When we look at the long term in exposure to flame retardants, it’s possible that people with less money will be less likely to buy new furniture and will still be retaining older furniture that might not be in good shape,” says Asa Bradman, an environmental health scientist at the University of California (UC), Berkeley, School of Public Health. He points out that these disparate exposures come on top of what are often higher exposures to outdoor air pollutants, closer residential proximity to waste treatment facilities and landfills, and higher levels of stress associated with poverty.^17^^,^^46^

## Disposal of Treated Items

According to sources in the furniture industry and manufacturers, furniture has an average of three owners each for 10–15 years, Blum says. This suggests that couches containing PentaBDE and other flame retardants could be in use for decades. Brenda Eskenazi, who directs the Center for Environmental Research and Children’s Health at the UC Berkeley School of Public Health, says her concern over what will happen to any padded furniture she might donate to a secondhand store leaves her in an ethical quandary.

“I’m still sitting on a couch that is contaminated,” she says. If she donates what is otherwise a perfectly good couch that contains chemical flame retardants, “who is going to take it but a person who can’t afford to buy a new couch?” she asks.

**Figure:**
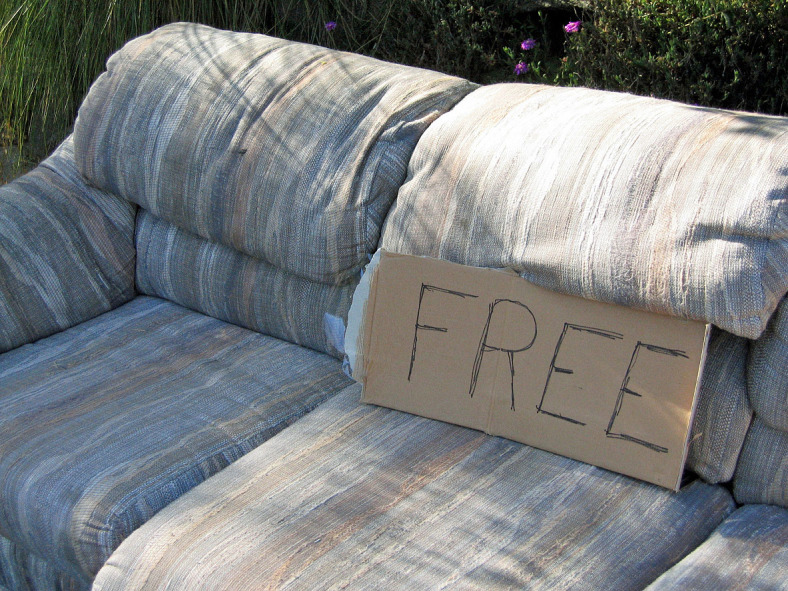
The benefits of a market-based approach to removing flame retardants from foam items will trickle down slowly to lower-income populations; people with less money are more likely to hold on to older products in which the foam is breaking down. This economic reality may help explain findings that lower-income homes tend to have higher levels of flame retardants in house dust. © Rob A. Johnston/Walkabout Wolf Photography/Getty

Blum has similar concerns. “For the whole eight years I’ve been dealing with this issue, I’ve been asking, what are we going to do with all of those toxic couches?” she says.

La Guardia says the way bulky items such as couches are handled by municipalities may provide people with the incentive to pass them along. “In some urban and suburban areas, municipalities charge to haul away your old sofa to the city dump. Charitable organizations will pick it up for free and sell it,” he says. The cost associated with taking items to the landfill can also encourage people to dump their old furniture illegally.

At present, the main option for disposing of old furniture and other foam products is landfills. Because over 50% of U.S. trash ends up in landfills—adding up to an estimated 135 million tons in 2012, according to the U.S. Environmental Protection Agency (EPA)^47^—these sites are likely already a major repository for flame retardants in the United States.

California places no restrictions on disposal of furniture in landfills. Because PBDEs are not considered hazardous waste, there is no mandate that they be deposited in hazardous waste landfills, giving the state’s Department of Toxic Substances Control no oversight of their disposal, says Russ Edmondson, a spokesman for the department. There also is no requirement that the air or water emitted by landfills be tested for the presence of any flame retardants, according to the California state officials interviewed for this article.

## Fugitive Flame Retardants

The extent to which landfill-related toxic exposures contribute to adverse health effects in people remains unknown.^48^ However, there is evidence that flame retardants do escape landfills. In Europe, elevated levels of flame retardants and other persistent compounds were measured in air near a landfill,^49^ and research on people living near and working in landfills in Nicaragua has indicated very high exposures to PBDEs.^50^ Studies have also shown that PentaBDE congeners can escape landfills via leachate.^51^

Landfills are a suspected source of increasing levels of PentaBDE congeners in air samples collected from remote sites around the Great Lakes by researchers at the Indiana University School of Public and Environmental Affairs.^52^ The levels of the flame retardants around Chicago and Cleveland dropped during the period when the concentrations at the remote sites appeared to be increasing. Ron Hites, the lead researcher on the Indiana University team, stresses that his group needs more data to confirm whether the concentrations are indeed increasing. If this trend is confirmed, he says landfills will be a potential exposure source worth investigating.

Other flame retardants used with polyurethane foam, such as TDCPP and some components of Firemaster 550, are more volatile than the PentaBDE they replaced,^30^ so they may be more likely to migrate out of landfills, says Martin Scheringer of ETH Zurich. TDCPP is also at least 1,000 times more soluble in water than BDE-47, a major constituent of PentaBDE.^27^^,^^53^

**Figure:**
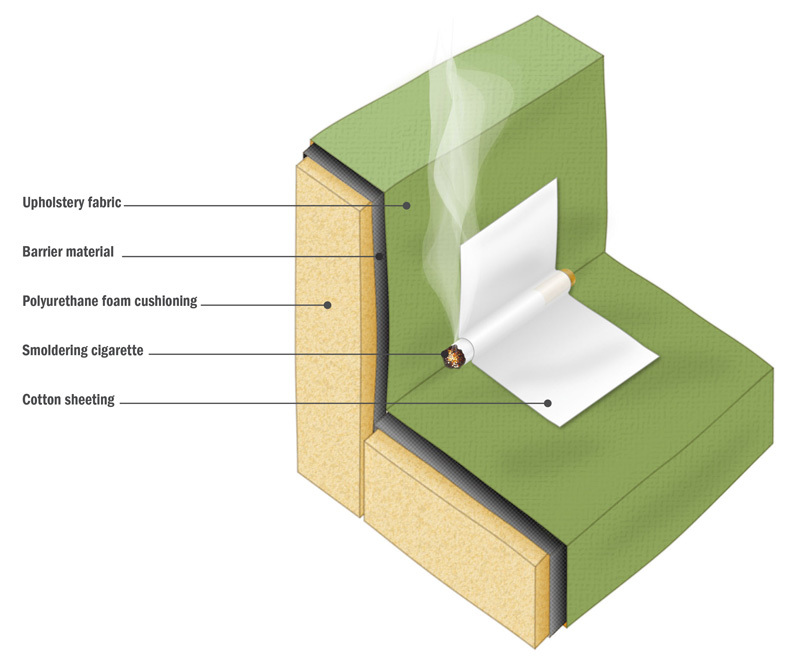
California’s earlier flammability standard required that foam be able to withstand an open flame applied for 12 seconds. The revised standard, TB117-2013, requires that furniture components pass an updated smolder test. In this test, a lit cigarette is placed on the surface of the material being tested. Materials fail if charring occurs on multiple samples more than 1.8 inches in any direction from the lit tip. The emphasis of the new standard is on stopping ignition of external materials to prevent the foam inside from burning. TB117-2013 does not forbid the use of chemical flame retardants. However, flame retardants are not needed to pass the test; many fabrics suitable for upholstery are inherently smolder-resistant, or manufacturers can place a smolder-resistant barrier material between the upholstery and the foam. © janewhitney.com

More research is needed in all these areas, researchers interviewed for this article agree. Rob Hale of the Virginia Institute of Marine Sciences has received funding from the EPA and the National Oceanic and Atmospheric Administration to investigate conditions under which flame retardants from different polymers, including polyurethane, may leach into water. This may provide insights into what happens inside landfills, he says.

Scheringer says decades of monitoring show that compounds such as the pesticide DDT and polychlorinated biphenyls, which have chemical properties similar to PBDEs, can volatilize and move around, sometimes over long distances, even though their high molecular weights would hypothetically limit their transport and make them likely to stay in place.^54^^,^^55^^,^^56^ This suggests that a percentage of the PentaBDE and other flame retardants that are added to landfills will escape, he says. Even if it’s only a fraction of a percent, the amounts liberated may be significant, given the amounts of these chemicals used, agree Scheringer and Åke Bergman of the Swedish Toxicology Sciences Research Center and Stockholm University. Another potential route of exposure for people living near or downwind of landfills is through consumption of food grown at home, Scheringer adds.

Improper disposal of products containing polyurethane foam presents another way in which flame retardants can be released into the environment. An abandoned piece of furniture may be a significant source of flame retardant emissions to the local environment, says Hale. “If it is abandoned and the foam degrades and contains flame retardants, the potential for it to be released locally is immense,” he says. “If it is in good shape and picked up rapidly, in a matter of days, it is not likely to be a significant source.”

Bergman argues strongly in favor of monitoring landfills to identify the extent to which flame retardants and other chemicals are emitted into the air and water. But he acknowledges that the levels of emissions from landfills are likely to be significantly lower than indoor emissions of flame retardants used in furniture,^9^ and therefore less of a direct health threat.

**Other Options for Consumers**

Two relatively new options are available to people who are concerned about the presence of flame retardants in unlabeled furniture and other foam products. First, anyone can “biopsy” their item and learn what it contains via Duke University’s free foam testing service.^59^ From there, individuals can decide if they want to keep the item or dispose of it.

The Green Science Policy Institute has launched a second option in the San Francisco Bay Area. In 2014 the institute began a pilot program known as the Safer Sofa Foam Exchange in which consumers can swap their old furniture foam for new foam that doesn’t contain flame retardants. Blum says the old cushions exchanged so far are being saved in a warehouse for research projects on responsible disposal of the foam.

The cost of the swap is an estimated $45–95 per cushion.^60^ “That’s very little compared to the potential health costs from the harm of the PentaBDE on kids’ IQs, and cancer, infertility, and immune problems. But the health costs don’t get factored into the equation,” Blum says. She has applied for a grant to develop strategies to bring such a foam exchange service to low-income communities for a reduced cost. But extending the program to stores that sell secondhand furniture “turned out to be very complicated,” Blum says, and her efforts in that area have been stymied.

**Figure:**
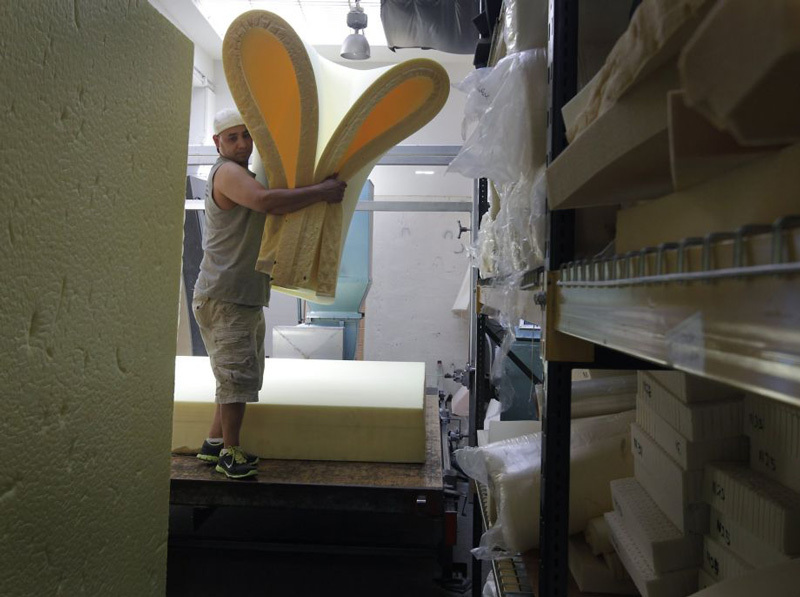
Foam and upholstery professionals in the San Francisco Bay Area are participating in the Safer Sofa Foam Exchange, in which consumers can swap existing cushion foam for new foam without added flame retardants. At an estimated cost of $45–95 per cushion, it’s cheaper than buying a new sofa. © Paul Chinn/San Francisco Chronicle

## Toward Solutions

The National Science Foundation is funding a workshop later this year aimed at identifying methods for disposing of and breaking down flame retardants in foam and plastics. UC Berkeley and the nearby Green Science Policy Institute, a science and policy organization, are currently planning the event, which will include scientists, regulators, and companies that have developed or are developing such methods.

At present, there aren’t many known options, says Don Lucas of the Lawrence Berkeley National Laboratory, who helped plan the workshop. He says that a technology based on incineration or other thermal techniques may prove to be the best alternative, but it must be carefully controlled to avoid unwanted by-products. Bergman points out that incineration is widely used in his native Sweden and elsewhere in Europe to manage municipal solid waste.

The current reality, of course, is that most Americans’ homes, vehicles, and workplaces still contain flame retardants, most likely a complex mixture of them. But studies have demonstrated that frequent handwashing may reduce human exposures to PentaBDE congeners and other flame retardants.^32^^,^^57^

Aimin Chen, an epidemiologist at the University of Cincinnati College of Medicine’s Environmental Health department, suggests that prospective mothers and other concerned individuals vacuum with a HEPA filter and use a wet cloth to dust furniture to reduce exposures. The nonprofit Environmental Working Group has developed a guide with further recommendations, such as replacing foam items that are misshapen and breaking down, keeping the covers of these items intact, and avoiding older foam items that are not completely encased in protective fabric.^58^

Not all these strategies have been conclusively shown to help reduce exposures to flame retardants. But even if they do, the discarded wash water, vacuum bags, and dust cloths will still transfer PentaBDE and other flame retardants out of the home, into the waste stream, and, eventually, into the environment. Birnbaum, Blum, and many others interviewed for this feature therefore urge manufacturers to continue moving toward safer chemicals for use in furniture and other applications with the potential for human exposure.

**Other Sources of Disparate Exposures**

Courtney Carignan, then at Boston University’s School of Public Health, was the lead researcher in a project that showed elite gymnasts can be exposed to high levels of flame retardants through their training equipment.^61^ For instance, the “foam pits” that gymnasts use to pad their landings during practice are filled with uncovered polyurethane blocks, which may leach flame retardants more quickly than upholstered furniture. Carignan says gymnasts aren’t the only ones who use foam pits—skiers, snowboarders, and other athletes use them, too. The pits are also a popular feature at many of the indoor trampoline parks that are popping up all over the United States.^62^

Flame retardants are an environmental justice issue for the gymnastics community for a number of reasons, says Carignan; less affluent gyms and schools will have more difficulty replacing the expensive specialized equipment and will be more likely to use secondhand equipment for fall protection, she explains. Carignan founded the nonprofit Gymnast Flame Retardant Collaborative, through which she runs a website and testing program to raise awareness among gymnasts and gym owners.^63^

Mark La Guardia of the Virginia Institute of Marine Science recently completed an analysis of air samples from four gymnastics studios, where he found equipment dating back to the 1996 Olympics.^64^ His testing showed that levels of flame retardants in the gyms’ air were several times higher than in the homes of coaches who worked in the gyms. “The studios’ owner had no idea that [the equipment] contained flame retardants and would probably not have exchanged it for years to come,” La Guardia says.

Other groups who can be disproportionately exposed to flame retardants in foam include carpet installers,65 people who work with recycled foam,^65^ firefighters (exposed to fumes from burning products),^66^ and those who dismantle and recycle discarded electronics.^67^^,^^68^

**Figure:**
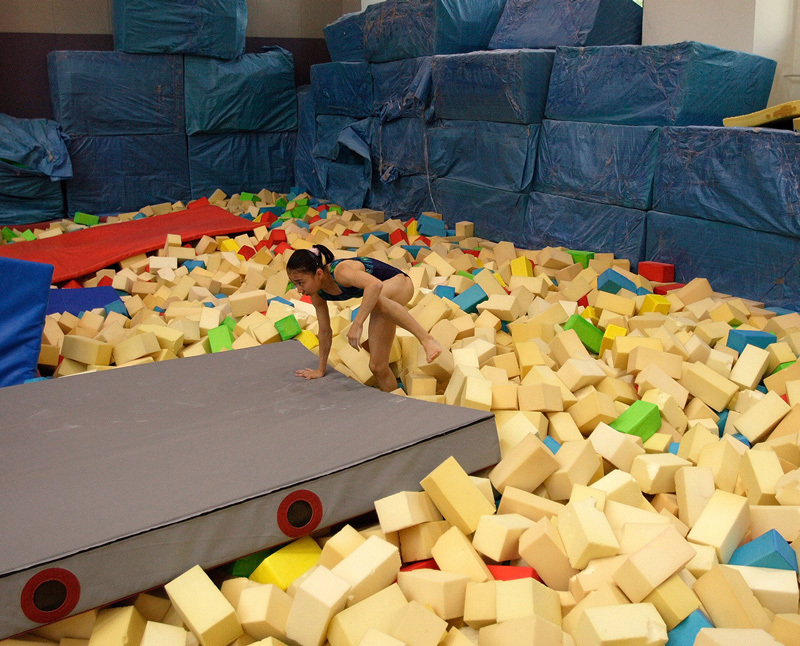
The bare blocks used in “foam pits” may leach flame retardants more quickly than fabric-covered foam items. Studies to date suggest that use of specialized equipment can result in especially high exposures to flame retardants among gymnasts and other athletes who train indoors. Foam pits have also become a popular feature at indoor children’s parks. © Reuters/Andrew Wong
